# Rapid microwave assisted RAFT synthesis of amphiphilic HEMA-co-AMPS copolymers for high performance Cu^2+^ and Cr^6+^ removal from water

**DOI:** 10.1038/s41598-026-41634-9

**Published:** 2026-03-27

**Authors:** Amany Gaffer, A. Ebada, Alshiama Refaat Alawady

**Affiliations:** 1 Petroleum Application Department, Egyptian Petroleum Institute (EPRI), Cairo, 11727 Egypt; 2 Egyptian Petroleum Institute (EPRI), Cairo, 11727 Egypt; 3 Polymers Lab, Petrochemicals Department, Egyptian Petroleum Research Institute, Cairo, Egypt

**Keywords:** Microwave-assisted synthesis, RAFT polymerization, HEMA-co-AMPS copolymers, heavy metal removal, Cu^2+^, Cr^6+^, Water treatment, Chemistry, Environmental sciences, Materials science

## Abstract

An amphiphilic HEMA-co-AMPS copolymer was synthesized through microwave-assisted RAFT polymerization using a rapid and energy-efficient approach. Comprehensive characterization confirmed successful copolymer formation, high structural stability, and the presence of abundant negatively charged sulfonate groups. The copolymer exhibited excellent adsorption performance toward Cu^2+^ and Cr^6+^ ions, achieving maximum capacities of 165 mg g⁻¹ and 115 mg g⁻¹, respectively, within ≤ 3 h. Adsorption followed a pseudo-second-order kinetic model, while equilibrium data were best described by the Langmuir model for Cu^2+^ and the Freundlich model for Cr^6+^. Thermodynamic analysis indicated spontaneous adsorption, with Cu^2+^ uptake occurring through an endothermic process and Cr^6+^ uptake proceeding exothermically. The copolymer retained more than 87% of its initial adsorption capacity after multiple cycles, demonstrating strong reusability. Overall, these findings highlight microwave-assisted RAFT polymerization as an efficient and sustainable strategy for producing high-performance polymeric adsorbents for water treatment applications.

## Introduction

Heavy metal contamination of water bodies has become a critical global concern due to rapid industrialization, mining activities, electroplating processes, and chemical manufacturing. Copper (Cu^2+^) and hexavalent chromium (Cr^6+^) are among the environmentally significant heavy metal pollutants because of their toxicity, non-biodegradable nature, and strong tendency to bioaccumulate in living organisms^1–3^. Exposure to elevated concentrations of these ions may cause severe neurological, hepatic, renal, and carcinogenic effects. Consequently, the removal of such contaminants from wastewater has become an urgent environmental and public health priority. In response, regulatory agencies worldwide have established strict discharge limits to mitigate their release into natural water bodies^1,4,5^.

In the present study, Cu^2+^ and Cr^6+^ ions were selected as representative model pollutants due to their environmental prevalence and contrasting chemical characteristics Cu^2+^ represents a typical divalent cation commonly detected in industrial effluents, whereas Cr^6+^ exists as highly toxic and mobile oxyanionic species in aqueous environments. Assessing both a cationic and an anionic metal ion enables a comprehensive evaluation of the amphiphilic HEMA-co-AMPS copolymer toward contaminants of differing charge states, thereby demonstrating its broad adsorption versatility.

Conventional removal methods, including chemical precipitation^6–9^, ion exchange^10–12^, membrane filtration^13–16^, and solvent extraction^17–19^, suffer from several limitations, such as high operational costs, secondary waste generation, limited selectivity, and reduced efficiency at low metal concentrations. Consequently, there is a pressing need to develop advanced adsorbent materials with high affinity, tunable surface functionality, and operational simplicity.

Polymeric adsorbents have emerged as particularly attractive materials for heavy metal remediation due to their structural versatility, tunable surface chemistry, and mechanical stability^20–22^. By incorporating functional groups such as carboxyl, hydroxyl, amide, and sulfonate moieties, polymeric materials can bind metal ions through electrostatic interactions, coordination, chelation, and hydrogen bonding. In this context, amphiphilic copolymers containing both hydrophilic and ionic segments offer enhanced performance in aqueous systems by improving water compatibility and increasing the density of accessible active sites^23,24^.

2-Hydroxyethyl methacrylate (HEMA) is a hydrophilic monomer that introduces hydroxyl groups, enhancing water dispersibility and contributing to hydrogen bonding and coordination interactions. Meanwhile, 2-acrylamido-2-methylpropane sulfonic acid (AMPS) contains strongly acidic sulfonate groups that remain ionized over a wide pH range, providing a high density of negative charges capable of binding metal ions. Copolymerization of HEMA and AMPS therefore enables the fabrication of multifunctional adsorbents with synergistic binding capabilities and improved adsorption efficiency^25^.

However, conventional free- radical polymerization often produces polymers with broad molecular weight distributions and limited control over architecture and functionality. Reversible addition–fragmentation chain transfer (RAFT) polymerization has emerged as a controlled radical polymerization technique that enables regulation of molecular weight, dispersity, and copolymer composition, which is essential for designing reproducible and efficient adsorbents.

Despite its advantages, conventional RAFT polymerization typically requires long reaction times and significant energy input. Microwave-assisted polymerization has recently gained attention as a rapid and energy-efficient alternative due to its volumetric heating mechanism, which accelerates reaction kinetics and improves homogeneity. Integration of microwave irradiation with RAFT polymerization provides a promising strategy to combine molecular control with fast and energy-efficient synthesis. Nevertheless, this approach remains insufficiently explored for the development of amphiphilic copolymers specifically designed for heavy metal removal.

Unlike conventional thermally initiated RAFT polymerization, which typically requires several hours to reach sufficient conversion, microwave-assisted RAFT polymerization enables rapid and homogeneous volumetric heating, accelerating initiator decomposition and the RAFT exchange process. This approach reduces reaction time and energy consumption while preserving functional group integrity. Compared with previously reported AMPS-based adsorbents synthesized via conventional thermal or free-radical routes, the present strategy offers improved synthesis efficiency and more uniform distribution of active sites, contributing to rapid adsorption kinetics and stable reusability.

This work aims to (i) develop a rapid and energy-efficient microwave-assisted RAFT synthetic route for HEMA-co-AMPS copolymers, (ii) systematically investigate the effects of reaction parameters on copolymer formation, and (iii) evaluate the adsorption performance toward Cu^2+^ and Cr^6+^ ions through kinetic, isotherm, and mechanistic studies. The findings provide new insights into the structure–function relationship of amphiphilic copolymers and demonstrate a sustainable pathway for designing high-performance polymeric adsorbents for wastewater treatment applications.

## Experimental

### Materials

2-Hydroxyethyl methacrylate (HEMA, 97%), 2-acrylamido-2-methylpropane sulfonic acid sodium salt (AMPS, ≥ 98%), 4-cyano-4-(phenylcarbonothioylthio) pentanoic acid (CPADB, 97%), and potassium persulfate (KPS, ≥ 98%) were received from Sigma Aldrich (USA) and used directly without further purification. CPADB was first dissolved in a minimum volume of ethanol to ensure its homogeneous dispersion before addition into the aqueous monomer mixture. Copper (II) sulfate pentahydrate (CuSO_4_·5H_2_O) and potassium dichromate (K_2_Cr_2_O_7_) were used to provide Cu^2+^ and Cr^6+^ ions, respectively. Deionized water was used throughout the studies, and nitrogen gas was used for purging dissolved oxygen during polymerization.

### Microwave Assisted RAFT Polymerization Mechanism and Procedure

The microwave-assisted RAFT polymerization of HEMA and AMPS was conducted in an aqueous medium using CPADB as the chain- transfer agent and KPS as the radical initiator. Under microwave irradiation, KPS rapidly decomposes into sulfate radicals (SO4·^−^​), which initiate polymerization by adding monomer units to form growing macroradicals (P·). These macroradicals undergo reversible addition to the C=S group of the CPADB agent, establishing a dynamic addition-fragmentation equilibrium. This equilibrium is critical for suppressing termination and preventing the formation of structural defects, such as branching or accidental cross-linking, by ensuring controlled chain growth and narrow dispersity.

**Scheme 1:**
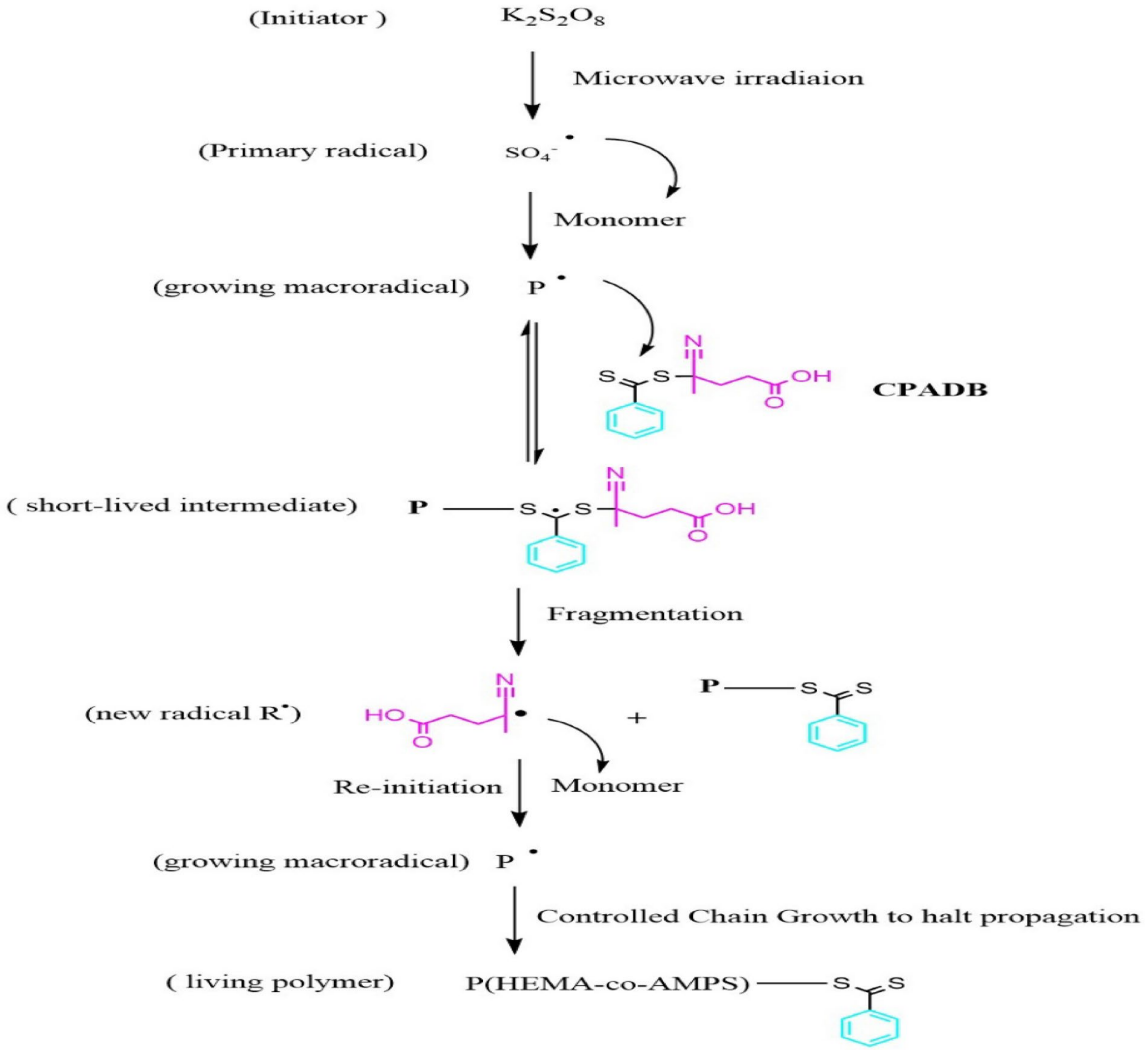

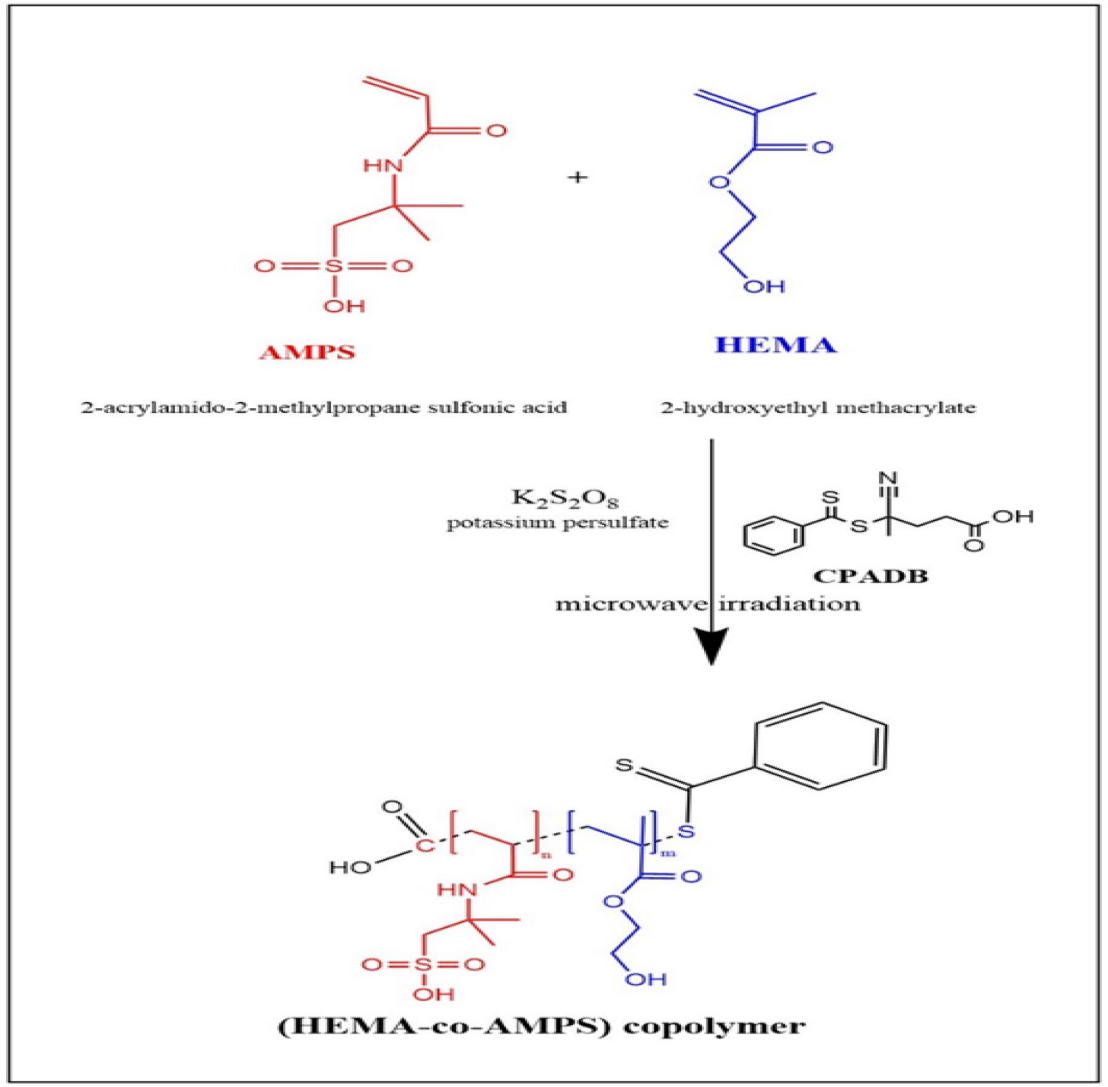
Proposed RAFT mechanism for microwave-assisted polymerization of HEMA and AMPS.

To further prevent structural degradation typically associated with long-term microwave exposure, the reaction parameters were strictly controlled. The polymerization used total monomer concentrations of 10–30 wt% in deionized water, with specific molar ratios of CPADB: monomer (1:80) and KPS: CPADB (0.2:1). The mixture was irradiated in a sealed vessel at 70–80 °C for a brief duration of 10–40 min at power levels up to 300 W. Unlike ionizing radiation, this controlled microwave energy provides rapid, volumetric energy transport that accelerates initiator decomposition and the RAFT exchange process without damaging the polymer backbone. At the conclusion of the irradiation period, the mixture was immediately cooled in an ice bath to halt propagation, ensuring the structural stability of the functional sulfonate and hydroxyl groups. The resulting copolymer was purified via dialysis for 48 h using a 3500 Da MWCO membrane and freeze-dried to obtain a dry powder as shown in Scheme 1^26^.

### Characterization techniques

The synthesized HEMA-co-AMPS copolymer was characterized to confirm its chemical structure, morphology, thermal stability, and surface charge using the following methods:

***Fourier Transform Infrared Spectroscopy (FTIR)***:

FTIR spectra were recorded in the 4000–400 cm^−1^ range using a Bruker Tensor 27 spectrometer to confirm copolymerization by identifying the characteristic functional groups present. FTIR analyses were also performed following the adsorption of Cu^2+^ and Cr^6−^ ions, where peak shifts due to metal binding could be identified^27,28^.

***Scanning Electron Microscopy (SEM)***:

The surface morphology of the freeze-dried copolymer was investigated by a JEOL JSM-6510LV microscope. A gold coating was sputtered onto samples before imaging at 5–15 kV. SEM imaging after metal adsorption was also conducted to study the structural and morphological changes resulting from metal binding^29,30^.

***Thermogravimetric Analysis (TGA)***:

Thermal stability was analyzed using a PerkinElmer TGA 4000 under nitrogen from 25 to 600 °C at a heating rate of 10 °C/min. TGA was performed on the pristine copolymer^31^.

***Zeta Potential Measurements***:

Zeta potential was measured at 25 °C using a zeta analyzer. Polymer suspensions (0.1 g/L) were adjusted to the desired pH values using 0.1 M NaOH or HCl. Measurements before and after metal adsorption were used to evaluate changes in surface charge associated with ion binding^32,33^.

Although RAFT polymerization is commonly associated with molecular weight and dispersity control, GPC/SEC analysis could not be reliably performed for the present highly sulfonated copolymer due to strong polymer–column interactions and solvent compatibility limitations. Therefore, quantitative molecular weight values are not reported and this is acknowledged as a methodological limitation.

### Heavy metal adsorption study

The adsorption performance of the synthesized amphiphilic copolymers toward Cu^2+^ and Cr^6+^ ions was evaluated using batch experiments. Polymer solutions were prepared at a concentration of 0.5 g/L and mixed with 50 mL of metal ion solutions at various initial concentrations (10–100 mg/L). The effects of solution pH, contact time, and initial metal ion concentration were systematically evaluated in separate experiments.

After equilibration, the solutions were filtered and analyzed using atomic absorption spectroscopy (AAS) to determine the residual metal ion concentrations^34^. The adsorption capacity (*q*, mg/g) was calculated using the following equation:

$$\hbox{q}=(\hbox{C}_{0}-\hbox{C}_{e})\,V/m$$

where *C*_0_ and *C*_*e*_ (mg/L) are the initial and equilibrium concentrations of metal ions, respectively; *V* (L) is the volume of the solution; and *m* (g) is the mass of the copolymer.

## Results and discussion

### FTIR analysis

**Fig. 1:**
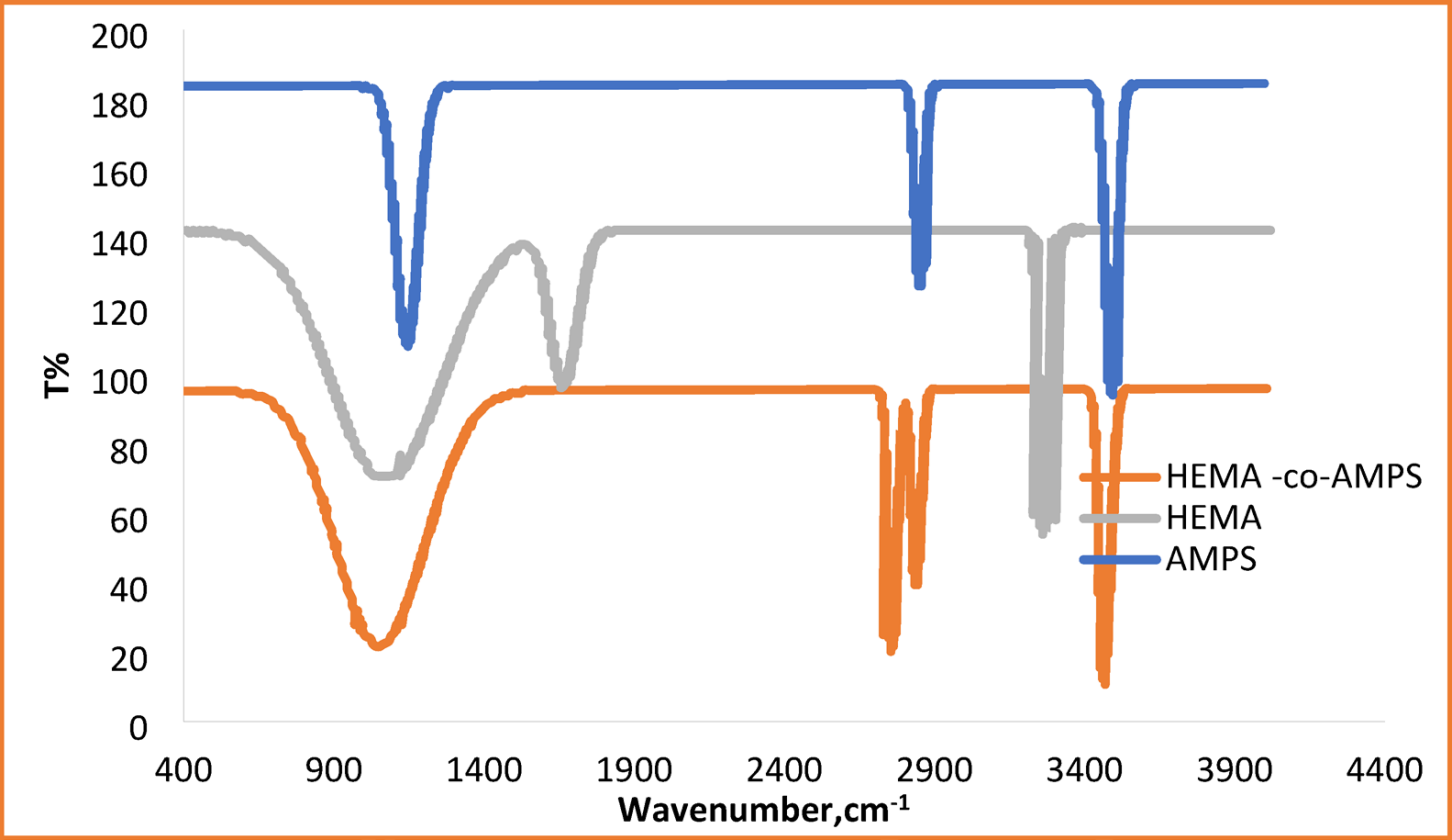
FTIR spectrum of HEMA, AMPS and HEMA-co-AMPS copolymer.

**Fig. 2:**
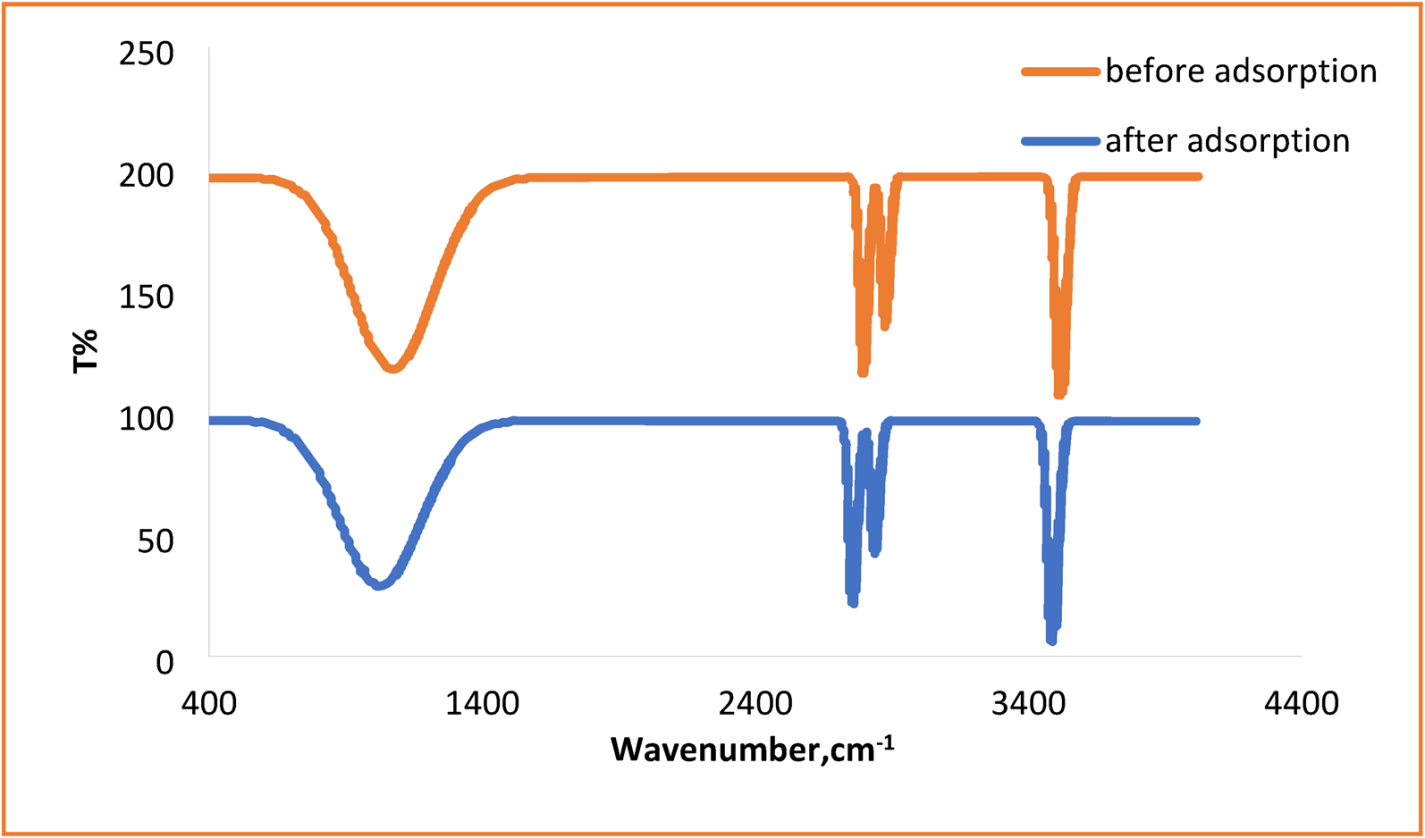
FTIR spectra of the HEMA-co-AMPS copolymer before and after Cu^2+^ and Cr^6+^ adsorption.

The copolymerization of HEMA and AMPS was confirmed, and the interaction between the HEMA-co-AMPS copolymer and Cu^2+^/Cr^6+^ ions was examined using FTIR spectroscopy. Figure 1 illustrates the FTIR spectra of HEMA, AMPS, and the HEMA-co-AMPS copolymer. In the region of 900–1200 cm^−1^, there is a broad and intense absorption band due to the overlapping sulfonate (SO_2_) stretching vibrations of AMPS and the CO stretching modes of HEMA. In the spectrum of the copolymer, this band is stronger than in pure homopolymers, which indicates the successful incorporation of both monomer units. Sharp absorption bands in the 3200–3600 cm^−1^ region correspond to OH stretching from HEMA and NH/OH stretching from AMPS. Aliphatic CH stretching bands appear at 2850–2950 cm^−1^. No bands corresponding to vinyl CH=CH stretching vibrations remain in the spectrum of the copolymer, indicating complete polymerization. Figure 2 shows the FTIR spectra of the copolymer before and after metal adsorption. The main intensity and shape changes in the spectra occurred after adsorption, affecting mostly the 900–1200 cm^−1^ region; thus, the sulfonate groups can be regarded as the primary adsorption sites via electrostatic interactions. Auxiliary involvement of hydroxyl and carbonyl groups through hydrogen bonding and weak electronic interactions is suggested by subtle variations in the 3200–3600 and 1650–1720 cm^−1^ regions, respectively. However, no significant changes in the shape or intensity of the bands of the aliphatic CH-stretching region were detected, which suggests that the polymer backbone remains structurally intact during the adsorption process. Accordingly, FTIR analysis indicates that sulfonate groups act as primary adsorption sites for metal ion binding, with additional contributions from hydroxyl and carbonyl functionalities through coordination and hydrogen bonding interactions^35^.

### SEM analysis

**Fig. 3:**
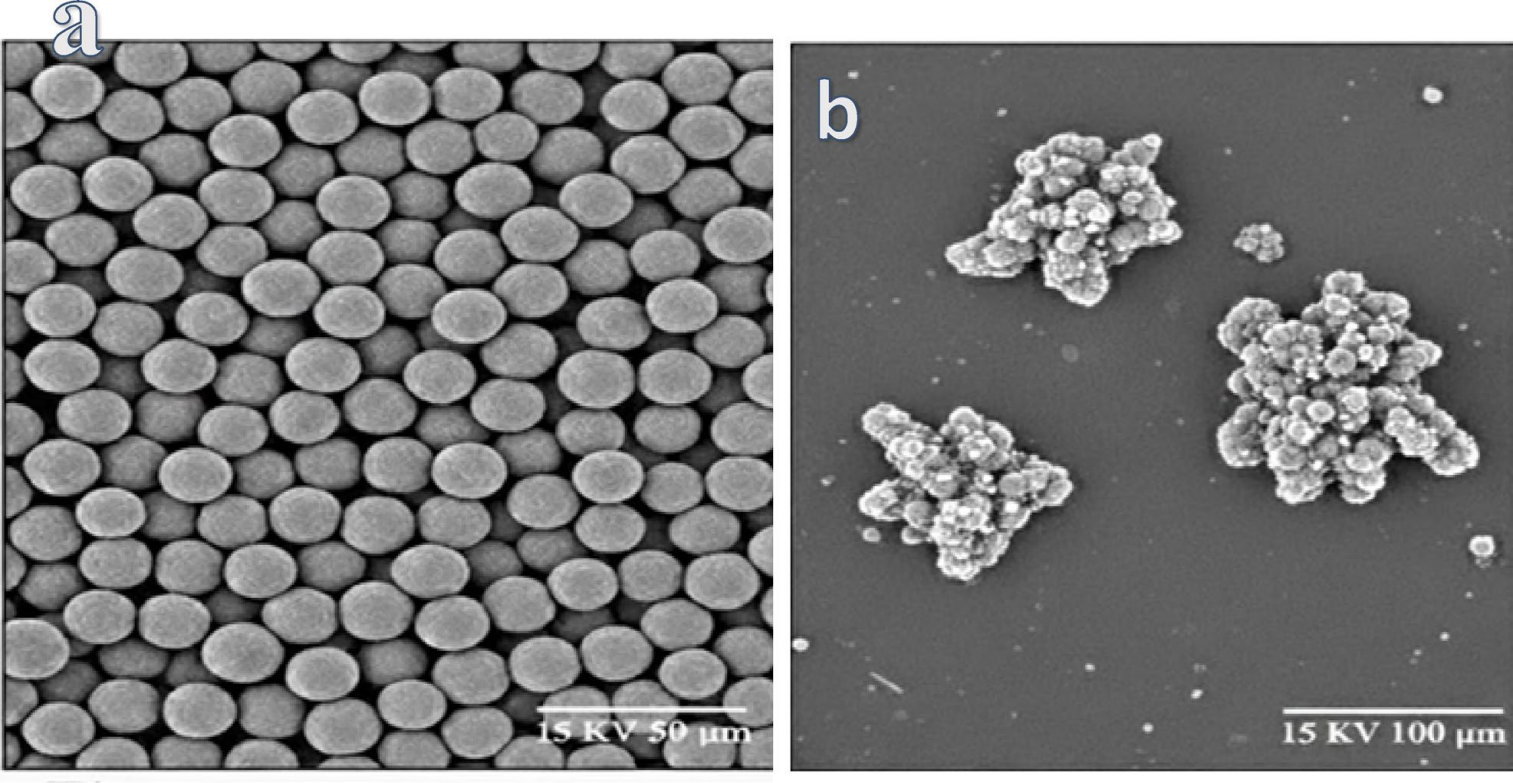
SEM images for (**a**) pristine copolymer & (**b**) copolymer after metal adsorption.

SEM micrographs of the pristine HEMA-co-AMPS copolymer and the metal loaded copolymer are presented in Fig. 3. The pristine material, as shown in Fig. 3a, is formed by uniformly distributed, almost spherical particles with smooth surfaces, narrow size distribution, indicating successful copolymer formation and good morphological homogeneity. The particles are well separated from each other, suggesting weak interparticle interactions in the absence of metal ions.

A clear change in surface morphology is observed after adsorption of Cu^2+^ and Cr^6+^ ions, as revealed by Fig. 3b. This includes the agglomeration of copolymer particles into irregular clusters with rough surfaces. Agglomeration may arise from metal ion bridging between functional groups like sulfonate (–SO_2_^−^), hydroxyl (–OH), and carbonyl (C=O), which facilitates interparticle crosslinking. Increased particle size and loss of spherical integrity upon adsorption demonstrate the immobilization of metal ions onto the copolymer matrix.

### Thermogravimetric analysis (TGA)

**Fig. 4:**
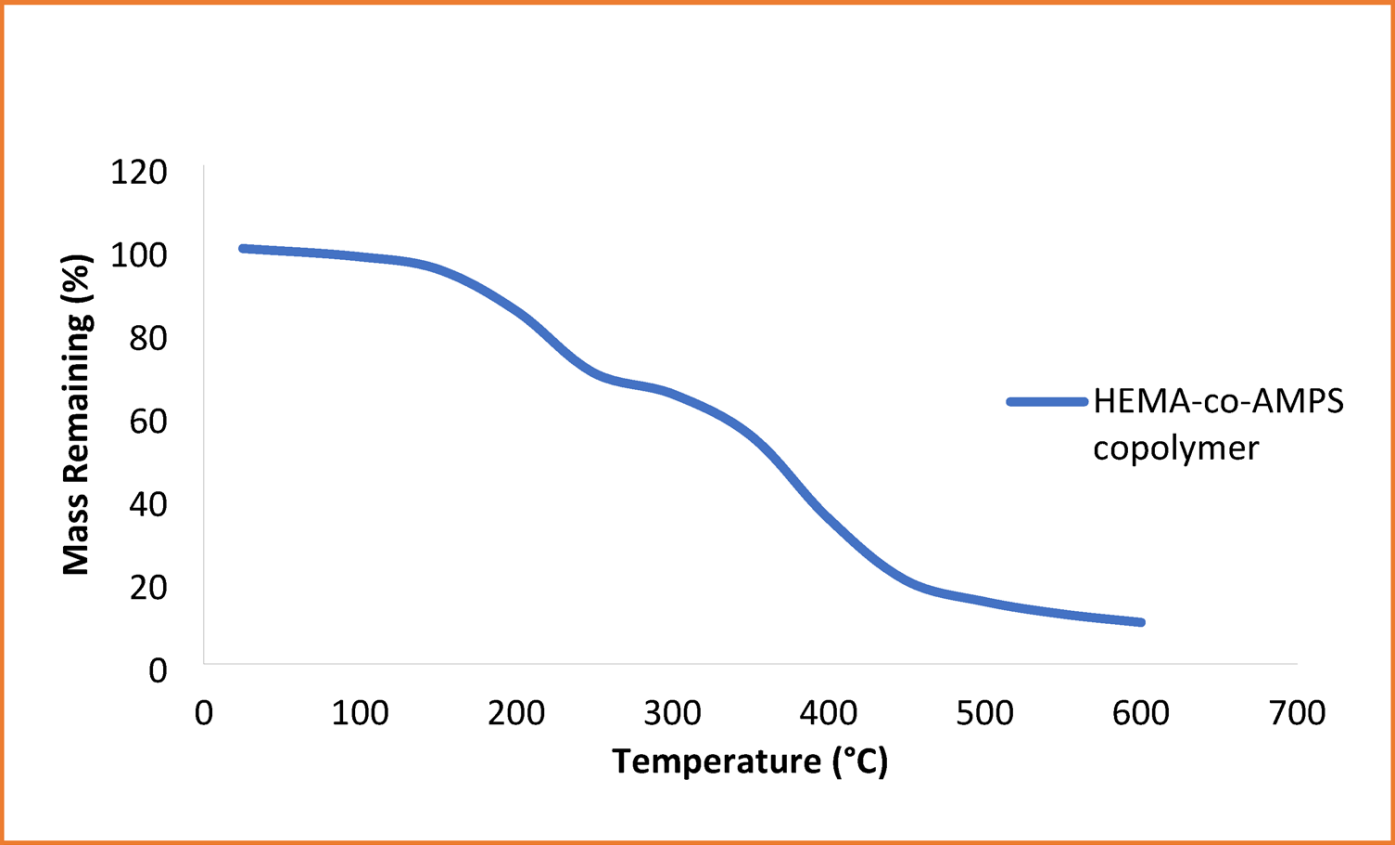
TGA of HEMA-co-AMPS copolymer.

Thermogravimetric analysis was conducted to study the thermal stability and decomposition behavior of the synthesized HEMA-co-AMPS copolymer. The TGA curve presented in Fig. 4 exhibits a multi-step degradation profile typical of hydrophilic, functionalized acrylic copolymers bearing hydroxyl, sulfonate, and amide groups. A slight initial mass loss below 150 °C is attributed to the evaporation of residual moisture physically retained within the polymer matrix. Hydrophilic OH and SO_2_^−^ functionalities facilitate water uptake that is typically reflected in this early loss region.

In the temperature range of 180–320 °C, there is a more pronounced weight loss which can be attributed to the cleavage of side-chain groups such as the decomposition of the HEMA ester moiety and partial breakdown of AMPS pendant functionalities. This is usually associated with the scission of weaker bonds such as C–O and C–N linkages. The major thermal degradation occurs between 320 and 480 °C, in which weight loss corresponds to the decomposition of the polymer backbone. During this stage, random chain scission of the carbon–carbon backbone occurs, with the elimination of sulfonate containing fragments, representing typical behavior reported in AMPS-based copolymers^36^.

The copolymer shows a small residual char above 500 °C, suggesting the presence of thermally stable sulfonated fragments, which carbonize under inert conditions. These findings confirm that the synthesized HEMA-co-AMPS copolymer possesses an adequate level of thermal stability for adsorption operations and further regeneration cycles, especially when moderate temperatures are used in drying and reusability experiments.

### Zeta potential analysis and adsorption mechanism

Zeta potential measurements were conducted to evaluate the surface charge characteristics of the HEMA-co-AMPS copolymer and to assess the contribution of electrostatic interactions during adsorption. The pristine copolymer exhibited a strongly negative zeta potential across the studied pH range, varying from − 45 mV at pH 2 to − 30 mV at pH 10. This negative surface charge is attributed to the dissociation of sulfonic acid groups (–SO_2_H) from the AMPS units, which remain ionized over a wide pH range.

**Fig. 5:**
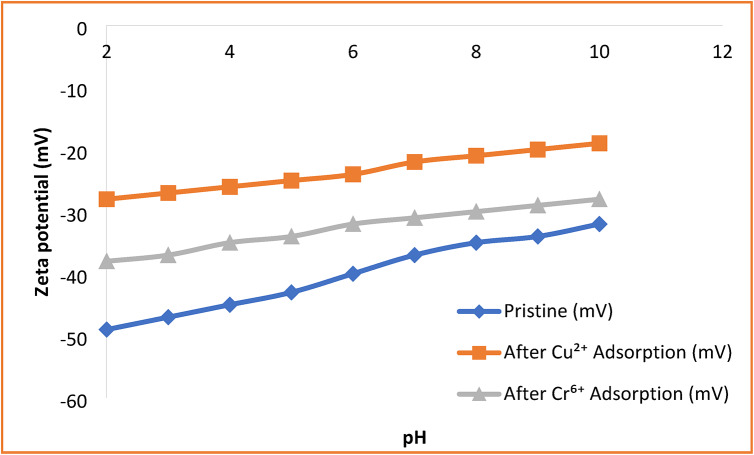
Zeta potential of HEMA-co-AMPS copolymer before and after metal adsorption.

After Cu^2+^ adsorption, the zeta potential shifted markedly toward less negative values, indicating significant electrostatic interaction between Cu^2+^ ions and the anionic sulfonate groups on the copolymer surface. In contrast, the shift observed after Cr^6+^ adsorption was comparatively limited, consistent with the predominantly anionic nature of chromate species (HCrO_4_^−^/CrO_4_^2-^) in aqueous solution. These results suggest that electrostatic attraction plays a major role in Cu^2+^ adsorption, whereas Cr^6+^ uptake is not primarily governed by classical electrostatic interactions. This interpretation aligns with the isotherm behavior observed for each metal ion Fig. 5^37^.

### Effect of pH on metal ion adsorption

**Fig. 6:**
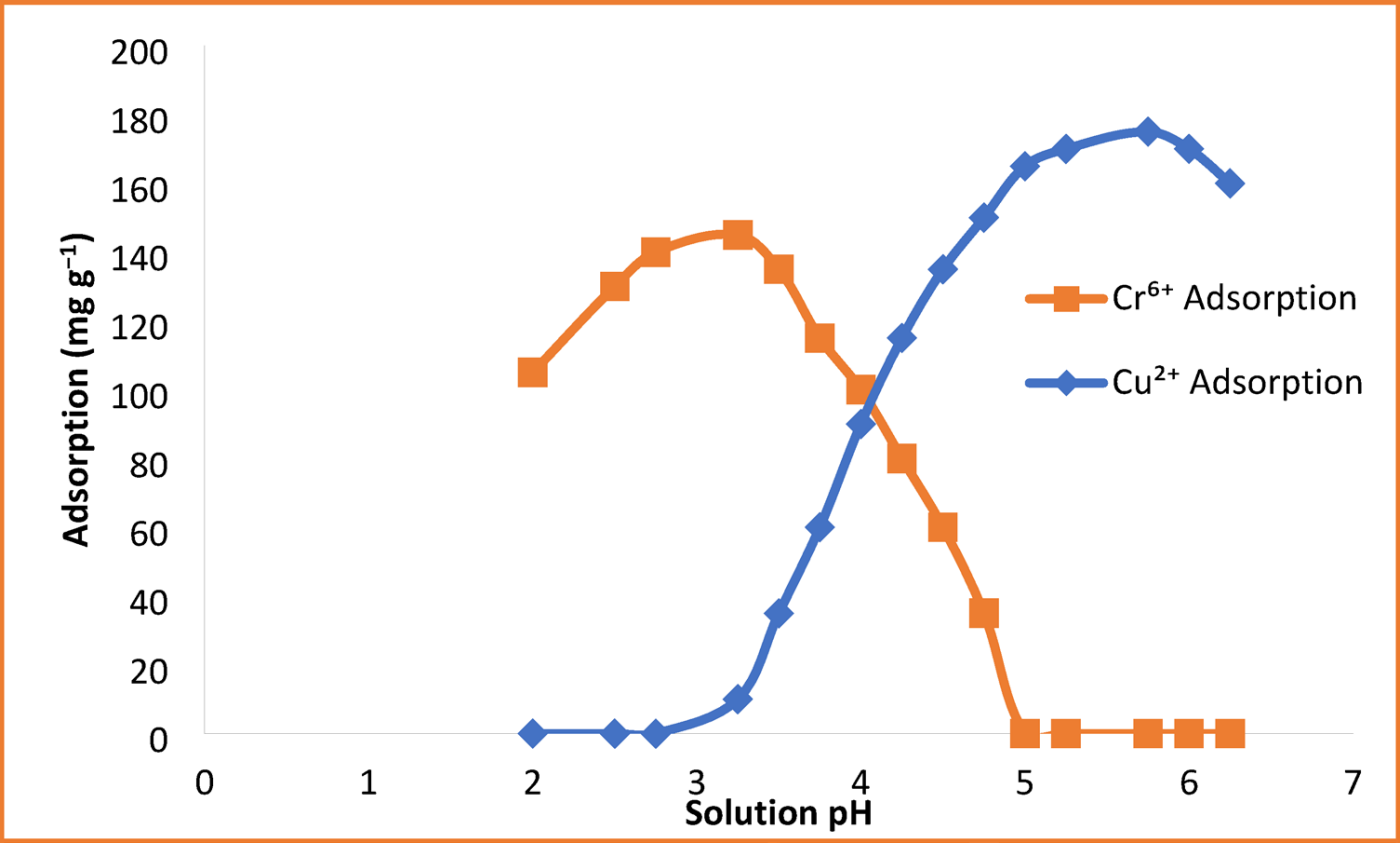
Effect of solution pH on the adsorption capacity of Cu^2+^ and Cr^6+^ ions.

Figure 6 shows that the solution pH exerts a strong impact on the adsorption of Cu^2+^ and Cr^6+^ onto the HEMA-co-AMPS copolymer. In the case of Cu^2+^ ions, as pH increases, the adsorption capacity increases progressively and reaches a maximum at pH 5.5. At low pH values, the excess of H^+^ ions compete with the Cu^2+^ ions for the same active sulfonate and hydroxyl binding sites, leading to reduced uptake. With the rise in pH, the deprotonation of functional groups strengthens the negative surface charge of the copolymer, enhancing electosatic attraction and coordination with Cu^2+^ ions.

Acidic conditions favor Cr^6+^ adsorption; it reaches its maximum capacity at around pH 3.0. This is because at low pH, Cr^6+^ species present mainly as anions such as HCrO_4_^−^ and Cr_2_O_7_^2−^, which interact with the protonated functional groups on the copolymer surface through electrostatic attraction and complexation. However, at higher pH values, the adsorption capacity drops sharply due to significant electrostatic repulsion between negatively charged Cr^6+^ species and the increasingly negative copolymer surface.

The pH dependent behavior confirms that electrostatic interactions play an important role in the mechanism of adsorption and emphasizes the selective removal ability of the amphiphilic copolymer for Cu^2+^ and Cr^6+^ under different pH conditions^38^.

### Effect of contact time

**Fig. 7:**
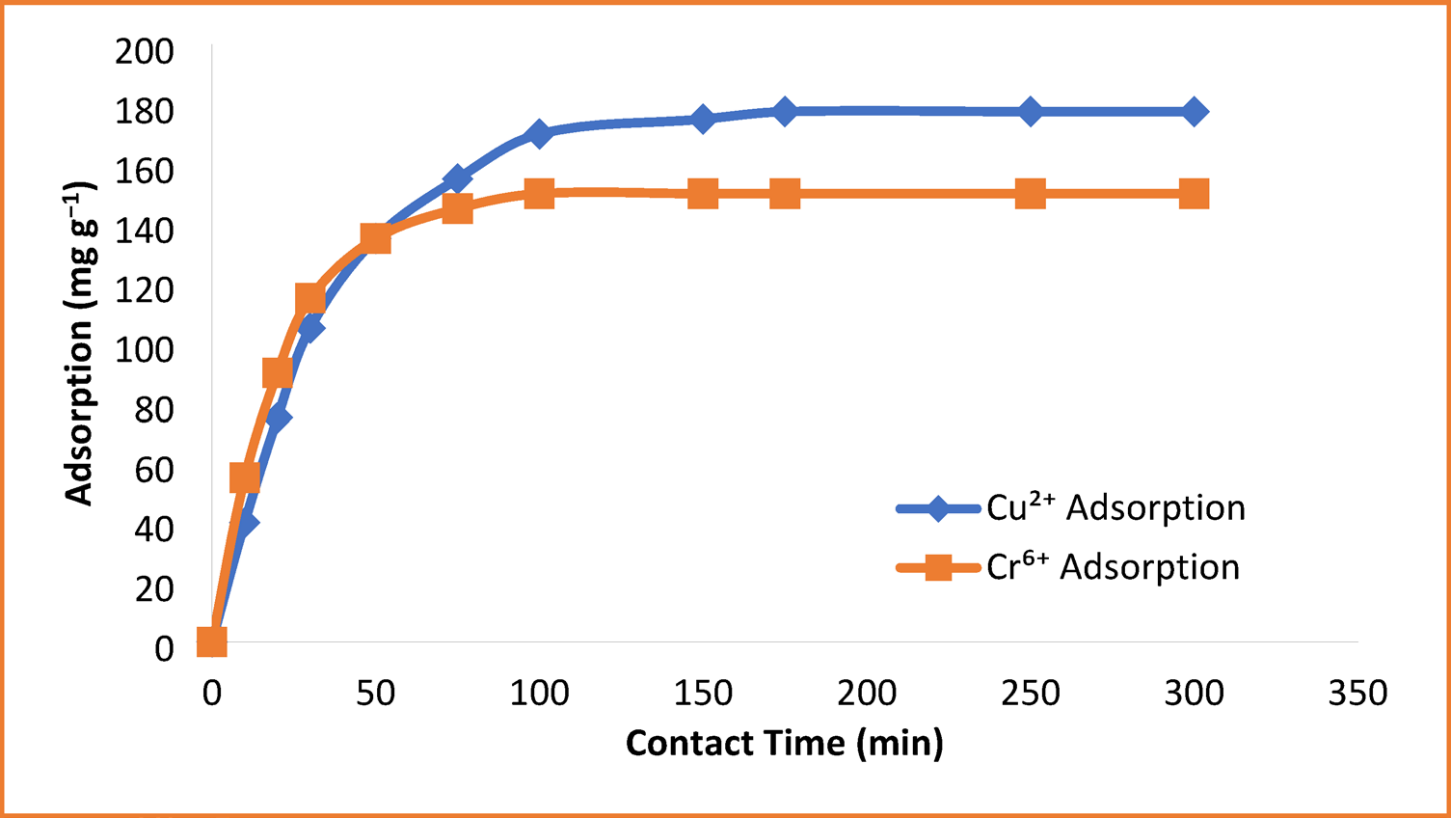
Effect of contact time on the adsorption of Cu^2+^ and Cr^6+^ ions.

Adsorption of both Cu^2+^ and Cr^6+^ ions increased rapidly in the initial contact period, reaching over 70% of their maximum capacities within the first 60 min as shown in Fig. 7. This is due to the ample availability of active adsorption sites on the surface of the copolymer, especially sulfonate and hydroxyl groups. The equilibrium is reached after around 120 min for Cr^6+^ and 180 min for Cu^2+^, after which no further considerable increase in adsorption capacity is observed. Such a plateau indicates saturation of active sites and attainment of adsorption-desorption equilibrium. These findings confirm that the HEMA-co-AMPS copolymer exhibits fast kinetics of adsorption, which is desirable in practical applications for water treatment^39^.

### Effect of Initial Metal Ion Concentration

**Fig. 8:**
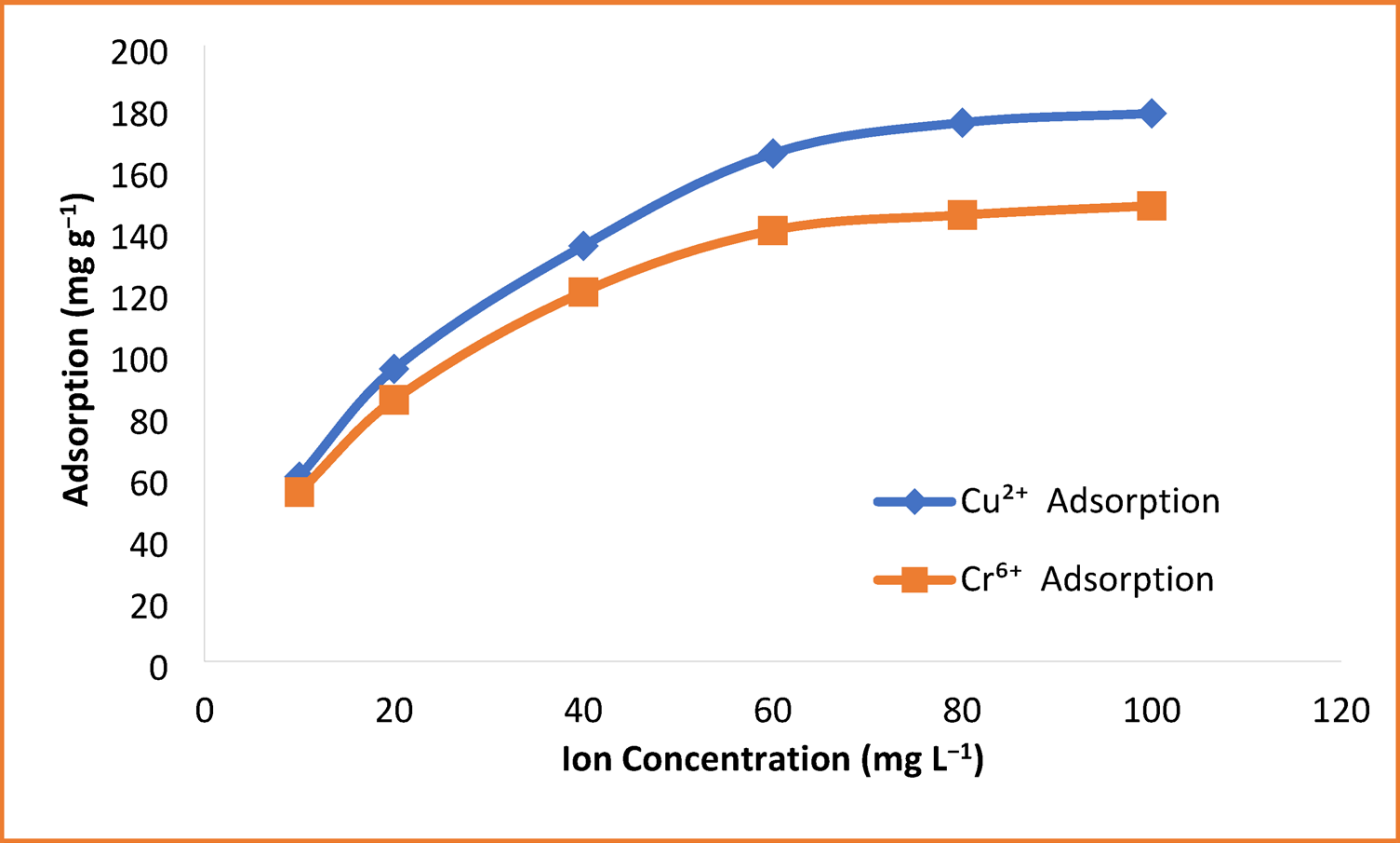
Effect of initial metal ion concentration on the adsorption capacity.

The effect of the initial metal ion concentration on adsorption performance of HEMA-co-AMPS copolymer was studied over a concentration range of 10–100 mg L^−1^, as shown in Fig. 8. In the case of Cu^2+^ and Cr^6+^, the adsorption capacity increases steadily with increasing initial metal concentration, reflecting a greater driving force for mass transport at higher concentrations. At relatively low initial concentrations, the number of adsorption sites available on the surface of the copolymer is greater than that of metal ions available, leading to a relatively lower uptake per unit mass^40^.

With an increase in the initial concentration, more metal ions interact with the active functional groups such as sulfonate, hydroxyl, and carbonyl moieties, and thus the adsorption capacity increases rapidly. At higher concentrations, the adsorption capacity reaches a plateau, indicating the progressive saturation of the available binding sites on the polymer surface. Cu^2+^ shows a higher maximum adsorption capacity compared to Cr^6+^ due to the strong electrostatic attraction and coordination affinity of divalent copper ions with the anionic sulfonate groups. For Cr^6+^, the slightly lower uptake is consistent with its predominant existence as anionic species in solution, leading to less favorable electrostatic interactions^41^.

These results confirm that the HEMA-co-AMPS copolymer maintains high adsorption efficiency across a wide range of contaminant concentrations and is particularly effective for treating wastewater containing elevated levels of heavy metals.

### Kinetic and Adsorption Isotherm Modeling

**Fig. 9:**
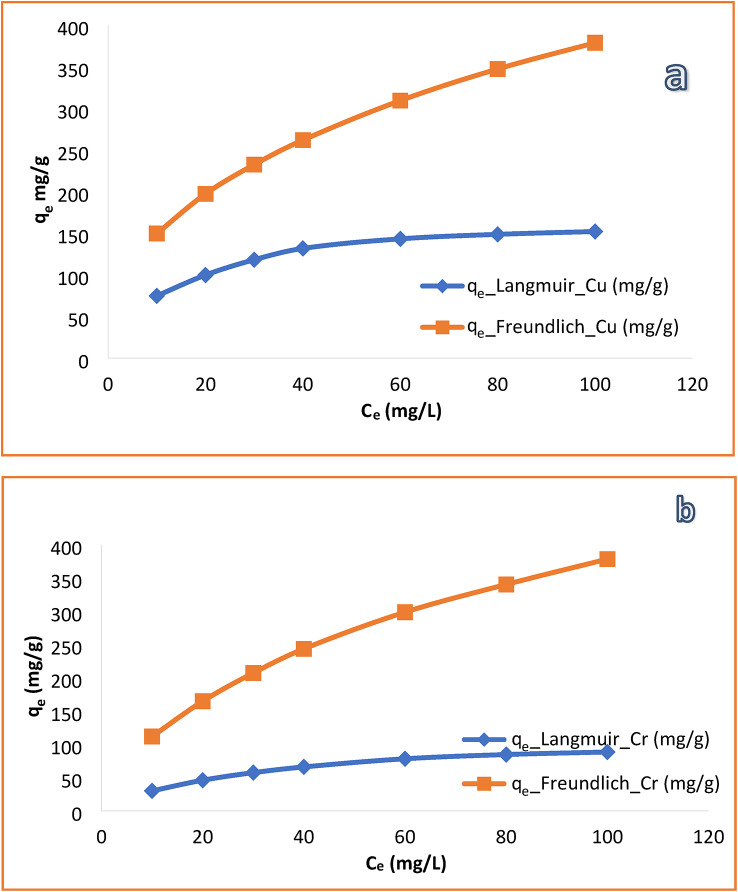
Nonlinear Langmuir and Freundlich isotherm fitting for the adsorption of (**a**) Cu^2+^ and (**b**) Cr^6+^ ions onto HEMA-co-AMPS copolymer at room temperature.

To quantitatively interpret the adsorption behavior of Cu^2+^ and Cr^6+^ ions onto the HEMA-co-AMPS copolymer, the experimental kinetic and equilibrium adsorption data were analyzed using pseudo-first order and pseudo-second order kinetic models, as well as Langmuir and Freundlich isotherm models. The corresponding fitting parameters are summarized in Table 1, and the nonlinear isotherm fittings are presented in Fig. 9a,b.

The pseudo-first- order and pseudo-second-order kinetic models are expressed as:

$$\begin{aligned}dq_{t}/dt&=k_{1}(q_{e}-q_{t})\\ dq_{t}/dt&=k_{2}(q_{e}-q_{t})^{2}\end{aligned}$$

where ${q}_{t}$(mg g^−1^) and ${q}_{e}$(mg g^−1^) are the adsorption capacities at time $t$and at equilibrium, respectively, and ${k}_{1}$(min^−1^) and ${k}_{2}$(g mg^−1^ min^−1^) are the kinetic rate constants.

The Langmuir isotherm model, which assumes monolayer adsorption on a homogeneous surface with a finite number of energetically equivalent adsorption sites, is expressed as:

$${q}_{e}=\frac{{q}_{max}{K}_{L}{C}_{e}}{1+{K}_{L}{C}_{e}}$$

where q_e_ (mg g^−1^) is the equilibrium adsorption capacity, q_max_ (mg g^−1^) is the maximum monolayer adsorption capacity, K_l_ (L mg^−1^) is the Langmuir constant related to adsorption affinity, and C_e_ (mg L^−1^) is the equilibrium metal ion concentration^42^.

The Freundlich isotherm model, applicable to heterogeneous surfaces and multilayer adsorption, is expressed as:

$${q}_{e}={K}_{F}{C}_{e}^{1/n}$$

where K_F_ is the Freundlich adsorption constant and 1/n represents adsorption intensity.

The pseudo second order model provided the best fit for both Cu^2+^ and Cr^6+^ adsorption (R^2^ > 0.99), indicating that chemisorption-related interactions dominate the adsorption process. Analysis of the equilibrium data revealed distinct isotherm behaviors for the two metal ions. Cu^2+^ adsorption followed the Langmuir model (R^2^ > 0.99), suggesting monolayer adsorption on relatively homogeneous binding sites. In contrast, Cr^6+^ adsorption was better described by the Freundlich model (R^2^ > 0.98; $1/n<1$), reflecting heterogeneous adsorption governed primarily by non-electrostatic interactions^43^.

The distinct fitting of Cu^2+^ to the Langmuir model and Cr^6+^ to the Freundlich model reflects their different interaction mechanisms with the HEMA-co-AMPS copolymer. Cu^2+^ adsorption followed the Langmuir model, indicating monolayer adsorption on homogeneous binding sites, whereas Cr^6+^ adsorption was better described by the Freundlich model, reflecting heterogeneous adsorption behavior^44^.

**Table 1:** Kinetic and adsorption isotherm parameters for Cu^2+^ and Cr^6+^ adsorption onto HEMA-co-AMPS copolymer.

Metal ion	Model type	Model	q_e_, exp/q_max_ (mg g^−1^)	q_e_, cal (mg g^−1^)	Rate/Isotherm constant	1/n	R^2^
Cu^2+^	Kinetic	Pseudo-first order	162.4	148.7	k_1_ = 0.021 min^−1^	–	0.931
Cu^2+^	Kinetic	Pseudo-second order	162.4	164.1	k_2_ = 1.8 × 10^−3^ g mg^−1^ min^−1^	–	**0.995**
Cr^6+^	Kinetic	Pseudo-first order	112.6	98.3	k_1_ = 0.017 min^−1^	–	0.918
Cr^6+^	Kinetic	Pseudo-second order	112.6	114.2	k_2_ = 2.4 × 10^−3^ g mg^−1^ min^−1^	–	**0.991**
Cu^2+^	Isotherm	Langmuir	165	–	K_L_ = 0.080 L mg^−1^	–	**0.992**
Cu^2+^	Isotherm	Freundlich	–	–	K_F_ = 58	0.41	0.961
Cr^6+^	Isotherm	Langmuir	115	–	K_L_ = 0.030 L mg^−1^	–	0.952
Cr^6+^	Isotherm	Freundlich	–	–	K_F_ = 32	0.54	**0.982**

Significant values are in bold.

### Thermodynamic analysis of Cu^2+^ and Cr^6+^ adsorption

The thermodynamic parameters governing the adsorption of Cu^2+^ and Cr^6+^ onto the HEMA-co-AMPS copolymer were evaluated to determine the spontaneity and thermal nature of the adsorption process. Adsorption experiments were conducted at three different temperatures (298, 308, and 318 K), and the distribution coefficient (Kc) was calculated using:

$$Kc=qe/Ce$$

The standard Gibbs free energy change (ΔG°) was determined from:

$$\varDelta G^{\circ}=-RTlnKc$$

**Fig. 10:**
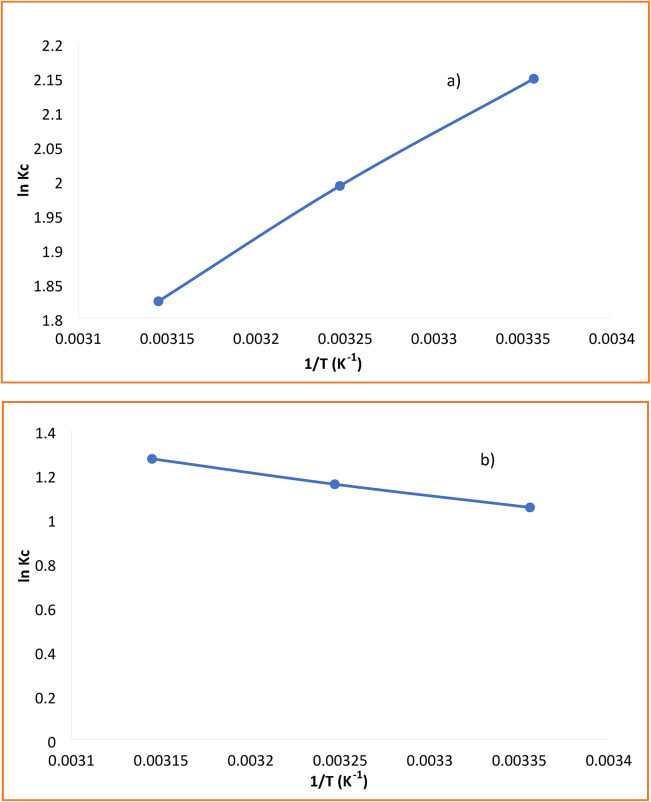
Van’t Hoff plots for (**a**) Cu^2+^ and (**b**) Cr^6+^ adsorption showing determination of ΔH° and ΔS°.

The enthalpy (ΔH°) and entropy (ΔS°) changes were obtained using the Van’t Hoff equation:

$$lnKc=\varDelta S^{\circ}/R-\varDelta H^{\circ}/RT$$

where R is the universal gas constant (8.314 J mol^−1^ K^−1^) and T is the absolute temperature (K). The linear Van’t Hoff plots (ln Kc vs. 1/T) for both metal ions are presented in Fig. 10, and the slope and intercept were used to calculate ΔH° and ΔS°, respectively. The linear regression analysis yielded high correlation coefficients (R^2^ > 0.99), confirming the reliability of the calculated thermodynamic parameters^25^.

As summarized in Table 2, the negative ΔG° values for both Cu^2+^ and Cr^6+^ confirm that the adsorption process is spontaneous over the studied temperature range. For Cu^2+^, ΔG° becomes more negative with increasing temperature, and the positive ΔH° value (+ 12.45 kJ mol^−1^) indicates an endothermic process. The positive ΔS° (+ 56.92 J mol^−1^K^−1^) suggests increased randomness at the solid–liquid interface during adsorption, likely associated with enhanced ion mobility and coordination interactions at elevated temperatures.

In contrast, Cr^6+^ exhibits a negative ΔH° (− 8.74 kJ mol^−1^, indicating an exothermic adsorption process. The slight increase in ΔG° values with temperature suggests reduced adsorption favorability at higher temperatures. The negative ΔS° (− 18.75 J mol^−^K^−1^) implies decreased randomness as chromate species become immobilized on the copolymer surface. These thermodynamic findings further support the distinct adsorption behaviors of Cu^2+^ and Cr^6+^ observed in the kinetic and isotherm analyses.

**Table 2:** Thermodynamic parameters for the adsorption of Cu^2+^ and Cr^6+^ onto HEMA-co-AMPS copolymer.

Metal ion	Temperature (K)	ΔG° (kJ/mol)	ΔH° (kJ/mol)	ΔS° (J/mol K)
Cu^2+^	298	− 4.52	+ 12.45	+ 56.92
	308	− 5.10		
	318	− 5.68		
Cr^6+^	298	− 3.15	− 8.74	− 18.75
	308	− 2.96		
	318	− 2.78		

### Reusability and regeneration performance

**Fig. 11:**
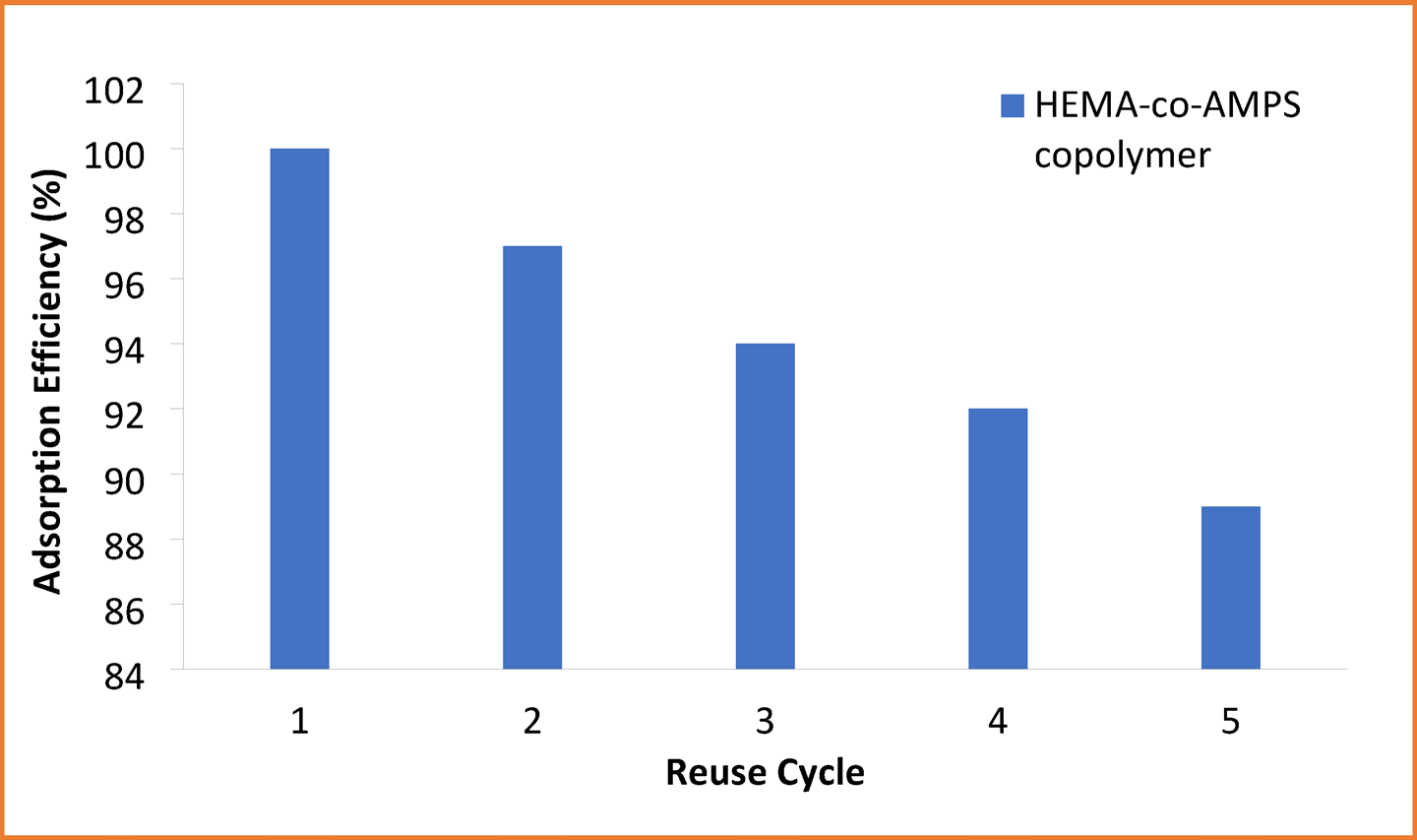
Reusability of the HEMA-co-AMPS copolymer for successive adsorption cycles.

To investigate the stability and practical applicability, the reusability of the HEMA-co-AMPS copolymer was examined over five consecutive adsorption desorption cycles. From Fig. 11, it can be observed that the adsorption capacity for both Cu^2+^ and Cr^6+^ decreases gradually with increasing cycle number. After five cycles, the remaining Cu^2+^ adsorption capacities was more than ~ 88%, and for Cr^6+^, ~ 87% of its initial adsorption capacity was still retained, showing good structural integrity and strong retention of active functional groups. The minor loss in adsorption performance may be due to the partial blockage of active sites or incomplete desorption of residual metal ions in the process of regeneration. Thus, according to these results, the synthesized copolymer possesses satisfactory reusability, confirming its potential for repeated use in the treatment of wastewater^45^.

### Comparison with reported polymeric adsorbents

The HEMA-co-AMPS copolymer performance with respect to adsorption capacity, equilibrium behavior, and regeneration stability was compared, as described above, with the reported polymeric adsorbents for Cu^2+^ and Cr^6+^ removal (Table 3). It is evident that the maximum adsorption capacities of the copolymer are around 165 mg g^−1^ for Cu^2+^ and 115 mg g^−1^ for Cr^6+^, reaching equilibrium in ≤ 3 h.

Moreover, the copolymer retains more than 87% of its original adsorption capability after several cycles of adsorption-desorption, demonstrating excellent structural stability. Unlike most reported adsorbents prepared by conventional thermal or multistep synthesis routes, the HEMA-co-AMPS copolymer benefits from the microwave-assisted RAFT synthesis, which enables rapid fabrication and controlled distribution of functional groups, contributing to fast kinetics and stable performances.

It should be noted that adsorption capacities reported in the literature can be strongly influenced by experimental conditions, including solution pH, initial metal ion concentration, adsorbent dosage, and temperature. Therefore, the comparison presented herein serves as a qualitative benchmark rather than a direct quantitative superiority claim. Under comparable operating conditions, the HEMA-co-AMPS copolymer demonstrates competitive adsorption capacity, rapid equilibrium attainment, and good reusability. These features, combined with the rapid microwave assisted RAFT synthesis, support its potential applicability in wastewater treatment.

**Table 3:** Comparison of HEMA-co-AMPS copolymer with reported polymeric adsorbents.

Adsorbent material	Target metal	q_max_ (mg g^−1^)	Equilibrium time	Reusability	Synthesis features	Ref.
Chitosan hydrogel	Cu^2+^	80–120	4–6 h	Moderate	Biopolymer, pH sensitive	^46^
Poly (acrylic acid) resin	Cu^2+^	100–150	3–5 h	Good	Conventional polymerization	^47^
Polymer/graphene composite	Cr^6+^	120–180	4–8 h	Moderate	Multi step, high cost	^48^
HEMA-co-AMPS (this work)	** Cu**^**2+**^	**≈ 165**	**≤ 3 h**	**Excellent (> 87%)**	**Microwave-assisted RAFT**	This work
HEMA-co-AMPS (this work)	** Cr**^**6+**^	**≈ 115**	**≤ 2 h**	**Excellent (> 87%)**	**Microwave-assisted RAFT**	This work

## Adsorption mechanism of Cu^2+^ and Cr^6+^ on HEMA-co-AMPS copolymer

**Fig. 12:**
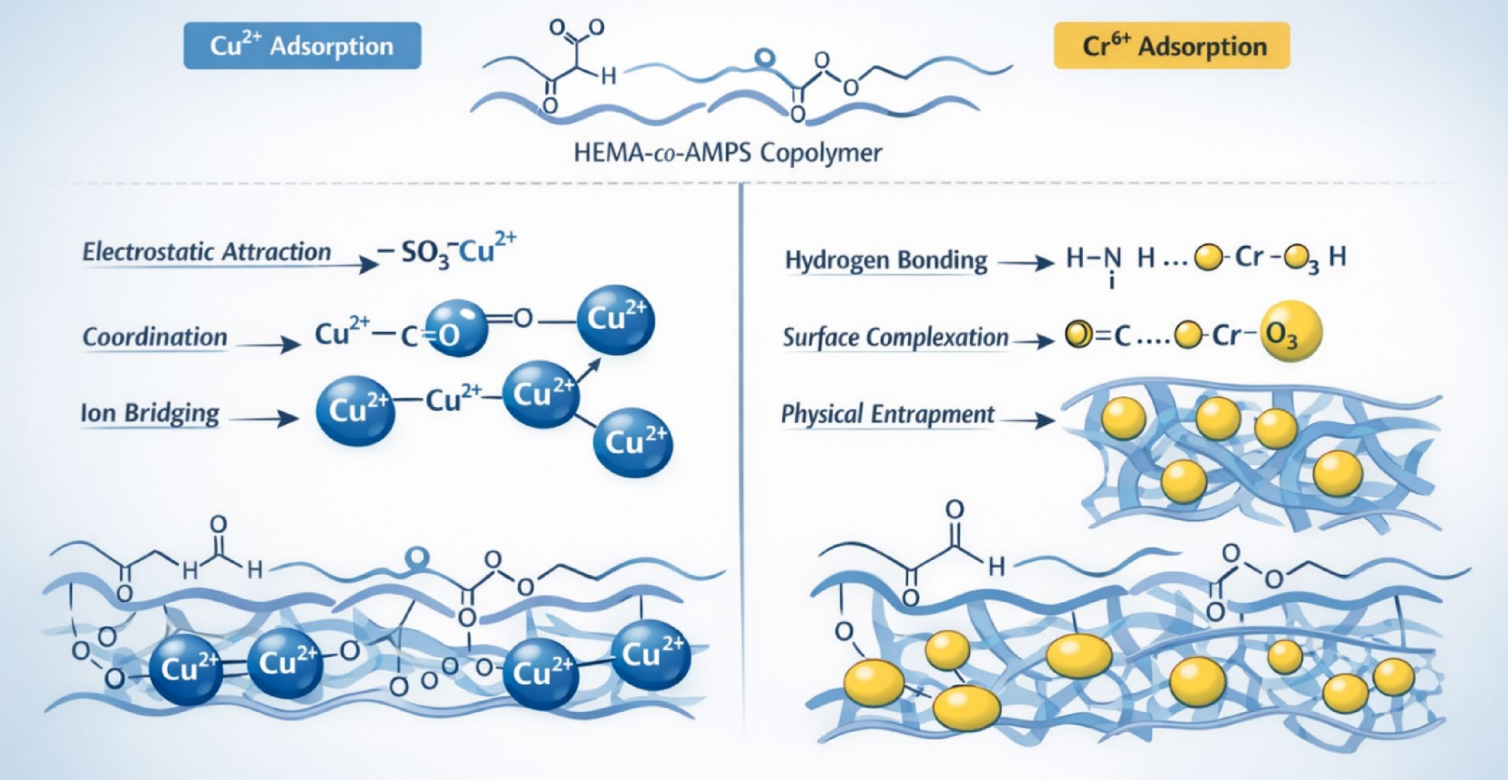
Proposed adsorption mechanism of (**a**) Cu^2+^ and (**b**) Cr^6+^ on HEMA-co-AMPS copolymer.

The adsorption mechanisms of Cu^2+^ and Cr^6+^ onto the HEMA-co-AMPS copolymer are illustrated in Fig. 12, highlighting the distinct interaction pathways responsible for metal uptake. The copolymer structure contains hydroxyl and carbonyl groups from HEMA units and strongly acidic sulfonate groups from AMPS units, which together provide multiple binding sites for metal ions.

For Cu^2+^, adsorption is primarily governed by electrostatic attraction between the divalent cations and negatively charged sulfonate groups on the polymer surface. Coordination interactions between Cu^2+^ and oxygen-donor atoms from carbonyl and hydroxyl functionalities further stabilize the adsorbed species. In addition, ion bridging between adjacent functional sites may occur, promoting uniform surface binding consistent with the Langmuir isotherm model behavior.

In contrast, Cr^6+^ exists predominantly as oxyanionic species such as HCrO_4_^−^ and CrO_4_^2-^ in aqueous solution. Because both chromate species and the copolymer surface carry negative charges, classical electrostatic attraction is limited. Therefore, Cr^6+^ adsorption occurs mainly through hydrogen bonding, surface complexation, and physical entrapment within the polymer network. These heterogeneous interactions agree well with the Freundlich isotherm model observed for Cr^6+^.

Overall, the adsorption process is governed by metal-specific interaction mechanisms, demonstrating that the HEMA-co-AMPS copolymer can effectively remove both cationic and anionic contaminants from aqueous environments.

## Conclusion

The amphiphilic HEMA-co-AMPS copolymer synthesized via microwave-assisted RAFT polymerization demonstrates outstanding performance for the removal of both Cu^2+^ and Cr^6+^ ions from aqueous media, combining rapid adsorption kinetics, high adsorption capacity, and excellent reusability. The material retained over 87% of its initial adsorption efficiency after repeated cycles, confirming its structural stability and practical applicability. Kinetic analysis establishes that adsorption follows a pseudo-second-order model, while equilibrium studies reveal Langmuir behavior for Cu^2+^ and Freundlich behavior for Cr^6+^, indicating metal-specific adsorption pathways.

Thermodynamic results confirm spontaneous adsorption for both ions, with Cu^2+^ uptake proceeding via an endothermic process and Cr^6+^ removal via an exothermic process. Mechanistic evaluation demonstrate that Cu^2+^ adsorption is dominated by electrostatic attraction and coordination interactions, whereas Cr^6+^ removal occurs mainly through hydrogen bonding, surface complexation, and physical entrapment within the copolymer network. These findings highlight the ability of the copolymer to efficiently capture both cationic and anionic contaminants through complementary interaction mechanisms.

This study establishes microwave-assisted RAFT polymerization as a rapid, energy-efficient, and sustainable platform for producing advanced functional adsorbents with controlled architecture and high performance. The developed HEMA-co-AMPS copolymer represents a robust and versatile material with strong potential for real-world wastewater treatment applications and provides a scalable strategy for designing next-generation polymeric adsorbents for environmental remediation.

## Data Availability

The datasets used and/or analyzed during the current study are available from the corresponding author on reasonable request .
